# Sodium Butyrate, a Histone Deacetylase Inhibitor, Exhibits Neuroprotective/Neurogenic Effects in a Rat Model of Neonatal Hypoxia-Ischemia

**DOI:** 10.1007/s12035-016-0049-2

**Published:** 2016-08-30

**Authors:** Malgorzata Ziemka-Nalecz, Joanna Jaworska, Joanna Sypecka, Rafał Polowy, Robert K. Filipkowski, Teresa Zalewska

**Affiliations:** 1 NeuroRepair Department, Mossakowski Medical Research Centre, Polish Academy of Sciences, 5 A. Pawinskiego Street, 02-106 Warsaw, Poland; 2 Behavior and Metabolism Research Laboratory, Mossakowski Medical Research Centre, Polish Academy of Sciences, Warsaw, Poland

**Keywords:** Neonatal hypoxia-ischemia, Histone deacetylase inhibitors, Sodium butyrate, Neuroprotection, Neurogenesis, Oligodendrocytes

## Abstract

Neonatal hypoxic-ischemic (HI) injury still remains an important issue as it is a major cause of neonatal death and neurological dysfunctions. Currently, there are no well-established treatments to reduce brain damage and its long-term sequel in infants. Recently, reported data show that histone deacetylase inhibitors provide neuroprotection in adult stroke models. However, the proof of their relevance in vivo after neonatal HI brain injury remains particularly limited. In the present study, we show neuroprotective/neurogenic effect of sodium butyrate (SB), one of histone deacetylase inhibitors (HDACis), in the dentate gyrus of HI-injured immature rats. Postnatal day 7 (P7) rats underwent left carotid artery ligation followed by 7.6 % O_2_ exposure for 1 h. SB (300 mg/kg) was administered in a 5-day regime with the first injection given immediately after the onset of HI. The damage of the ipsilateral hemisphere was evaluated by weight deficit. Newly produced cells were labeled with BrdU, at 50 mg/kg, injected twice daily for 3 consecutive days. Subsequent differentiation of the newborn cells was investigated 2 and 4 weeks after the insult by immunohistochemistry using neuronal and glial cell-lineage markers and BrdU incorporation. Finally, we performed several behavioral tests to evaluate functional outcome. In summary, SB led to a remarkable reduction of the brain damage caused by HI. Moreover, the application of this HDACi protected against HI-induced loss of neuroblasts and oligodendrocyte precursor cells, as well as against neuroinflammation. The observed neuroprotective action suggests that SB may serve as a potential candidate for future treatment of HI-evoked injury in neonates.

## Introduction

Neonatal hypoxic-ischemic encephalopathy (HIE) remains one of the most important causes of neonatal mortality and long-term neurological sequelae such as cerebral palsy, mental retardation, epilepsy, and spastic paresis [1–4]. Encephalopathy due to hypoxic-ischemic (HI) events derives from acute and unpredictable episodes of perinatal asphyxia. The interruption of blood supply to the brain leads to insufficient oxygen and glucose delivery and triggers a cascade of biochemical events including loss of energy, acidosis, excitotoxicity, elevation of intracellular calcium, induction of oxidative stress, inflammation, and apoptosis, culminating in extensive brain damage [2, 5]. Undoubtedly, brain damage following hypoxia-ischemia is a complex process and develops over several hours to days and by this, it provides an opportunity for therapeutic intervention in the sequence of intracellular processes induced by HI. However, despite the significant progress in knowledge relating the mechanism(s) underlying evolving brain injury, there are no well-established effective therapies to reduce brain damage and its long-term sequel in infants. The clinical application of promising neuroprotective agents has been truly restricted due either to inefficient or adverse effects.

The lack of expecting effective neuroprotective treatments for newborns suffering from HIE brings about increased interest in alternative therapies based on stem cells, all the more because neonatal HI brain injury stimulates a neurogenic response. Although postnatal neurogenesis has been reported to exist in many brain areas, it is seen most consistently in two regions-subgranular zone (SGZ) of the hippocampal dentate gyrus (DG) and the subventricular zone of the lateral ventricles (SVZ). The HI-induced enhanced proliferation of neural progenitors in these regions is followed by the migration of newly generated cells toward the injured brain areas, where they acquire the desired phenotype during the differentiation process, enabling the damaged brain area to be reconstructed [6–8]. However, it occurred that the endogenous repair processes do not resolve the brain damage, as the capacity of endogenous regeneration proved to be rather limited and insufficient for replacing the lost neurons [9, 10]. Thus, strategies are being sought to amplify the endogenous regenerative response by introducing molecules that might expand stem cell proliferation.

Recently, epigenetic agents have received considerable attention and have spurned justified hope as therapeutic approaches against acute brain injury. Several data published in the last decade provided significant evidence of neuroprotection obtained by histone deacetylase inhibitor (HDACi) administration after stroke in the mature brain. The neuroprotective effect was associated with a decrease in brain lesion, neurobehavioral improvement, and stimulation of neurogenesis [11–17]. A significant amount of data suggests that HDACis display protective properties also in a number of neuropathological conditions. Therefore, it may be postulated that treatment with these agents has emerged as an attractive therapeutic approach in in vivo models of neurodegeneration as well as in acute brain ischemia [16, 18–22].

Acetylation of histones is a key post-translational modulation of proteins responsible for the regulation of critical intracellular pathways. This process is carried out by two classes of nuclear enzymes, histone acetylases (HATs) and histone deacetylases (HDACs). Increased level of histone acetylation is associated with the release of condensed chromatin and enhanced gene transcription. Histone deacetylation, on the other hand, promotes chromatin condensation and gene silencing. Although histones are the most intensively investigated substrates, the activity of diverse non-histone proteins, such as transcription factors, signal transduction mediators, is also modified by HATs and HDACs [23, 24]. All these data imply that the disruption of acetylation homeostasis may have a serious impact on cellular functioning. This suggestion remains in line with decreased level of acetylated histones in neurodegenerative disorders as well as in brain ischemia insult [18, 22].

HDACis have been shown to promote neuronal differentiation of neural progenitor cells [25] via increased expression of proneural genes Ngn1, Math1, and P15 [26] in association with increased histone acetylation. Moreover, a number of drugs with HDAC inhibitory activity have been shown to protect mature neurons against acute brain injury [18, 27].

Despite mounting evidence concerning the role of HDACis in neuroprotective/neurogenic processes in the experimental ischemic model in adults, only a few studies addressed the impact of HDAC inhibitors upon post-HI neurogenesis in the immature brain [28–31]. Since many aspects of the evolving brain damage following hypoxic-ischemic insult, and thereby the efficacy of neuroprotectants differs between adults and neonates, extrapolating data obtained in the mature brain to neonates is generally unwise. Therefore, the aim of our study was to examine whether treatment with HDAC inhibitor–sodium butyrate has neuroprotective/neurogenic effects in a rat model of neonatal HI and, if so, to assess whether this neuroprotection is associated with the decreasing HI-induced functional deficits. Finally, based on previous reports indicating that growth factors released by brain ischemia-injured cells may be crucial for the neurogenic response in adult animals, we sought to determine whether endogenous BDNF is involved in the generation of new cells after hypoxic-ischemic injury. Our attention was primarily focused on the hippocampus which is vulnerable to HI damage and has the potential for neurogenesis.

## Materials and Methods

### Experimental Neonatal Hypoxia-Ischemia

All animal experiments were conducted according to a protocol approved by the Local Ethics Committee for Animal Experimentation. Cerebral hypoxia-ischemia was produced in 7-day-old (P7) Wistar rats of either sex by a permanent unilateral common carotid artery ligation, followed by systemic hypoxia [32, 33]. As was previously reported, the ligation alone does not decrease cerebral perfusion below critical levels, and the addition of hypoxia is required to cause brain infarct [34]. Briefly, pups were anesthetized with isoflurane (4 % induction, 2.0 % maintenance) carried by O_2_. Once they were fully anesthetized, a midline neck incision was made and the left common carotid artery was isolated, double ligated with surgical silk, and cut between two ligatures. The incision was then sutured with monofilament nylon. Sham-operated animals underwent the same surgical procedure without the ligation of the carotid artery. The time length of anesthesia lasted on average 5 min. After surgery, the rat pups were returned to their home cage for 1 h recovery. Hypoxia was induced by placing the animals in a chamber (35 °C) and subjecting them to a humidified mixture of 7.6 % oxygen in nitrogen for 1 h.

The undamaged hypoxic hemisphere, as well as age-matched sham-operated animals, served as controls. Pups from each litter were randomly assigned to 4 experimental groups (5 rats per group and time point): (1) control group (vehicle treatment) (2) control animals (SB treatment) (3) animals which underwent HI (vehicle treatment), (4) animals which underwent HI (SB treatment). Animals were sacrificed at specific time points (3, 6, 7, 9, 11, 14 or 28 days) after the injury.

### Drug Administration and Bromodeoxyuridine Labeling

Rats subjected to HI or sham-operated were treated with subcutaneous injections of sodium butyrate (SB, Sigma-Aldrich; 300 mg/kg body weight according to the published data [13, 14]) or vehicle (saline) starting immediately after hypoxic exposure and lasting 5 consecutive days.

Endogenous cell proliferation was determined by 5-bromo-2-deoxyuridine (BrdU) cell incorporation. BrdU (Sigma-Aldrich) dissolved in physiological saline was administered intraperitoneally (50 mg/kg per injection, in sterile 0.9 % NaCl plus 0.007 N NaOH). Two-injection paradigms were used. In the first paradigm, animals received a single dose of BrdU and were sacrificed 24 h after the injection. This procedure was used to determine the number of cells that incorporated BrdU during a 24-h period at a specific time point after HI (3, 6, 9, 11, 14 days). In other experiments, the animals received BrdU injections twice daily (12 h apart) for 3 consecutive days starting 4 days after the onset of hypoxia-ischemia. Animals in this group were sacrificed 14 and 28 days after the insult. This allowed us to determine the phenotype of newborn cells.

### Brain Injury Evaluation

Fourteen days after the insult (at postnatal day 21), the pups were anesthetized with 100 mg/kg ketamine combined with 10 mg/kg xylazine and decapitated, and the brains were dissected. The brain stem and cerebellum were removed from the forebrain. The two cerebral hemispheres were separated in the midline and weighed. The brain damage was assessed by the weight deficit (%) of the ipsilateral (injured) hemisphere relative to the contralateral (hypoxic) hemisphere [35].

### Tissue Preparation

At the scheduled time points, anesthetized animals were perfused transcardially first with phosphate buffered saline (PBS) followed by a fixative solution (4 % paraformaldehyde, PFA, in 0.1 M phosphate buffer, pH 7.4). The brains were removed and submerged in the same fixative solution for 4 h at 4 °C. Following postfixation, the brains were cryoprotected overnight in 30 % sucrose solution (in 0.1 M PBS), frozen rapidly using dry ice, and placed in −80 °C storage.

For biochemical analysis, animals were sacrificed through decapitation and the hippocampi or whole hemispheres were frozen on dry ice. All tissue samples were stored at −80 °C until used.

### Western Blot Analysis

Brain tissues were homogenized in RIPA lysis buffer (10 mM Tris-HCl pH 7.5 containing 150 mM NaCl, 1 % Nonidet P40, 0.1 % SDS, 1 % Triton X-100, PMSF 0.1 mg/ml) and a proteinase and phosphatase inhibitors cocktail (Life Technologies, 1:100). Lysates were clarified by centrifugation at 13,000 g for 10 min at 4 °C. The supernatant was collected and used for analysis of BDNF (as described below). Cell pellets were resuspended in RIPA lysis buffer. Protein concentrations were determined using a Bio-Rad DC™ protein assay kit (Bio-Rad) in the supernatant as well as in the pellet solution. Samples of the pellet (50 μg protein) were ran on 10–15 % SDS-PAGE gels and transferred onto nitrocellulose membranes (Amersham Bioscience). After blocking, membranes were probed with the polyclonal anti-acetyl H3 antibody (Millipore) and then incubated with horseradish peroxidase-conjugated secondary IgG antibodies (Sigma-Aldrich). Immunoblot signals were visualized using ECL chemiluminescence kit (GE Healthcare Life Sciences). To verify an equal loading of protein per line, the beta-actin antibody was used as an internal control for each immunoblotting. Semi-quantitative evaluation of protein levels detected by immunoblotting was performed by computer-assisted densitometric scanning (LKB Utrascan XL, Program GelScan). The level of protein immunoreactivity was determined by frequent analysis of multiple immunoblots.

### Immunohistochemistry

The following antibodies (source and final dilution) were used for tissue staining: sheep polyclonal anti-BrdU (Abcam, 1:500), mouse monoclonal anti-BrdU (Santa Cruz Biotechnology, 1:100), rabbit polyclonal anti-doublecortin (DCX; Cell Signaling 1:200), rabbit monoclonal anti-calbindin (Cell Signaling, 1:200), rabbit polyclonal anti-NG2 chondroitin sulfate proteoglycan (Millipore, 1:200), mouse monoclonal anti-oligodendrocyte marker (O4; Millipore, 1:200), mouse monoclonal anti-myelin basic protein (MBP; Millipore, 1:200), and rat monoclonal anti-ED1 (CD68) (AbD Serotec, 1:100).

Coronal cryostat sections of the brain (30 μm thick) were cut at the level of the dorsal hippocampus in serial order to create 10 series sections. Double fluorescent immunohistochemistry was performed on free-floating sections comprising the hippocampal formation. After blocking for unspecific reactivity, adjacent series of sections were stained for a specific cell-lineage marker. For detection of BrdU incorporation, DNA was first denaturated by incubation of sections with 2N HCl at 37 °C for 1 h and rinsed for 15 min in 0.1 M sodium tetraborate (pH 8.5) at room temperature. After blocking with 10 % normal goat serum in PBS containing 0.25 % Triton X-100 for 60 min and washing with PBS, sections were incubated with anti-BrdU overnight at 4 °C. Following the washing procedure, the primary antibodies were revealed by appropriate secondary FITC-conjugated (AlexaFluor, 1:500) antibodies for 60 min at room temperature and in the dark.

Differentiation of BrdU-positive cells was monitored with markers labeling neurons or oligodendrocytes at various stages of maturation (DCX, calbindin, NG2, O4, MBP) and microglia/macrophages (ED1). After BrdU staining, the brain-tissue sections were incubated with primary antibodies overnight at 4 °C. After being rinsed in PBS, the sections were exposed to secondary antibodies for 1 h at room temperature. Negative controls were processed in the same manner on adjacent sections but with the primary antibodies omitted.

Double labeling to determine the expression of lineage markers by BrdU expressing cells was verified using a confocal laser scanning microscope (LSM 780, Carl Zeiss, Germany) using a ×20 objective. A helium-neon laser (543 nm) was utilized in the excitation of Alexa Fluor 546, while an argon laser (488) was applied in the excitation of FITC.

The number of BrdU-positive cells in the entire DG area was assessed in an average of five hippocampal sections per animal. To avoid double counting, we did not analyze adjacent sections. Every section was evaluated using a computerized system, and the positive cells were displayed on a computer screen. All of the counting was performed using ImageJ 1.46 software.

### Quantitative Measurement of BDNF Protein Concentration

To estimate the amount of BDNF in lysates obtained from the hippocampi and hemispheres, the ChemiKine Brain Derived Neurotrophic Factor, Sandwich ELISA (Millipore) test was applied according to the supplier’s instructions. After performing the Sandwich ELISA assay, the plates were read at 450 nm using a spectrophotometric plate reader Fluorostar Omega (BMG LabTech).

### Behavioral Tests

Experimental animals were divided into three groups: control (C), *n* = 12; hypoxia-ischemia (HI), *n* = 11; and hypoxia-ischemia with sodium butyrate treatment (HI+SB), *n* = 12. During behavioral experiments, rats were kept in a 12-h light-dark cycle with water and food provided ad libitum. The experiments were done during the light phase of the cycle. Animal behavior was monitored at P33–83.

All testing and training were conducted by an observer blind to the treatment group in a sound attenuated room.

*Open field* (OF) was initiated at P33–34 and performed for 3 days. OF box dimensions were 55 × 55 × 50 cm. Rats were gently placed in the middle of the OF floor. The recording lasted for 15 min. After each trial, the apparatus was cleaned with 10 % ethanol solution. Animal behavior was recorded with Basler acA1300-60 GigE camera (Bassler AG, Germany) and scored using Ethovision XT 10 (Noldus Information Technology, Netherlands). For the analysis, OF floor was virtually divided into three zones: (i) border, 9.2 cm wide; (ii) middle, 9.2 cm wide and (iii) center square, 18.3 × 18.3 cm. The following parameters were measured: latency to the first entrance to a zone, frequency of entering zones, percent time duration in each zone, mean velocity, and total distance moved (comp. [36–38]).

*Rotarod* (accelerating Rota-Rod 7750,TSE systems, Germany) was started at P39–40 and conducted according to Karalis et al. [39]. On the first day only, the test was preceded by habituation, i.e., placing the rat on a stationary cylinder for 30 s and thereafter for 2 min with a constant low-speed rotation (4 rpm). Animals that fell from the rod were placed again on it until they were able to stay for 60 s. After at least 10-min rest, the animals were tested in accelerating conditions. The cylinder accelerated from 4 to 40 rpm in 300 s. The time of the trial was scored when the rat fell from the cylinder, spun with the cylinder 3 times consecutively without walking or reached a maximum of 500 s without falling. The device was cleaned with 10 % ethanol solution between animals. Each rat was scored once daily for 4 days.

*Grip test* (Bioseb BP, In Vivo research Instruments, France) was initiated at P46–47 and done for 2 consecutive days [40, 41]. To measure the forepaw grip strength of the rat, it was held by the trunk and the base of the tail. Then it was guided onto a metal grid with 90.5 cm square opening, attached to a force transducer, and encouraged to grab it by forepaws only. Then the animal was steadily pulled backwards until it lost hold of the grid. Three measurements in Newtons per rat were taken with at least 1 min of interval between trials to let the animal rest.

*Morris water maze* (MWM; comp. [42–44]) was started at P62. The pool was 150 cm in diameter, the water temperature was of around 25 °C, and it was dyed gray to discourage the animals from diving. A square platform (10 × 10 cm) was submerged 1.5 cm underneath the surface in the middle of one of the quadrants. Rats’ home cages were left in the pool room for at least 1.5 h to accommodate. For each trial, the rat was placed on the platform for 60 s, and then it was put, facing the walls of the pool, into the water at 1 of 4 starting positions. The positions were pseudo-randomly varied from trial to trial, with the restriction that in each series of 4 trials all 4 possible positions had to be used. The animal had 60 s to find the platform. In case of failure, the rat was guided to the platform where it stayed for 60 s. There were 2 trials per day for 6 days. On the seventh day, the probe trial was performed with the platform removed and the rats placed in the opposite quadrant. The trial lasted for 60 s; it was repeated a week later. Animal behavior was recorded and scored as in OF experiments. The visible platform test was performed in the same conditions, but the tank was covered around with curtains to remove visual cues of the room. The platform was marked with a flag and placed consecutively in 4 different positions. The rat was put into the water in the opposite side of the pool related to the platform. Time required for the animal to reach the platform was scored.

*Ultrasonic vocalizations* (USV) were evoked by tickling. The procedure was initiated at P81 with some of the above-mentioned animals (C, *n* = 6; HI, *n* = 6; HI+SB, *n* = 5). It consisted of 2 days of pre-training with a familiar experimenter and the test day. The procedure was the same during pre-training and the experiment. Namely, the rat was placed in the tickling cage (57 × 38 × 20 cm) with a high sensitivity condenser microphone hung 30 cm above the center of the cage floor. The rat waited for 30 s in the tickling cage before the procedure. Then it was tickled for 15 s firstly by rapid finger movement around the neck area and then by flipping the animal on its back and rapidly moving fingers on its belly. The next 15 s was spent by moving the experimenters hand around the cage and allowing the rat to chase it. This was repeated 4 times [45]. USV were automatically scored on the spectrogram (Hamming, frame size 100 %, overlap 50 %) with Avisoft SASLab Pro software.

### Statistical Analysis

GraphPad PRISM 5.0 software was used for the statistical analysis of the received data (excluding behavioral test results). Comparisons between animal groups were performed using one-way analysis of variance (ANOVA) with post hoc Bonferroni test for multiple comparisons. All values were expressed as mean ± SD. All data received from behavioral tests are represented as means with standard error of the means (SEM). The effects of behavioral experiments were analyzed with ANOVAs. Significant main effects or interactions were followed up with post hoc analysis (Duncan), where appropriate. The calculations were made using Statistica 7.1. (StatSoft Inc., 2005).

## Results

### Effects of Sodium Butyrate on Brain Damage

**Fig. 1:**
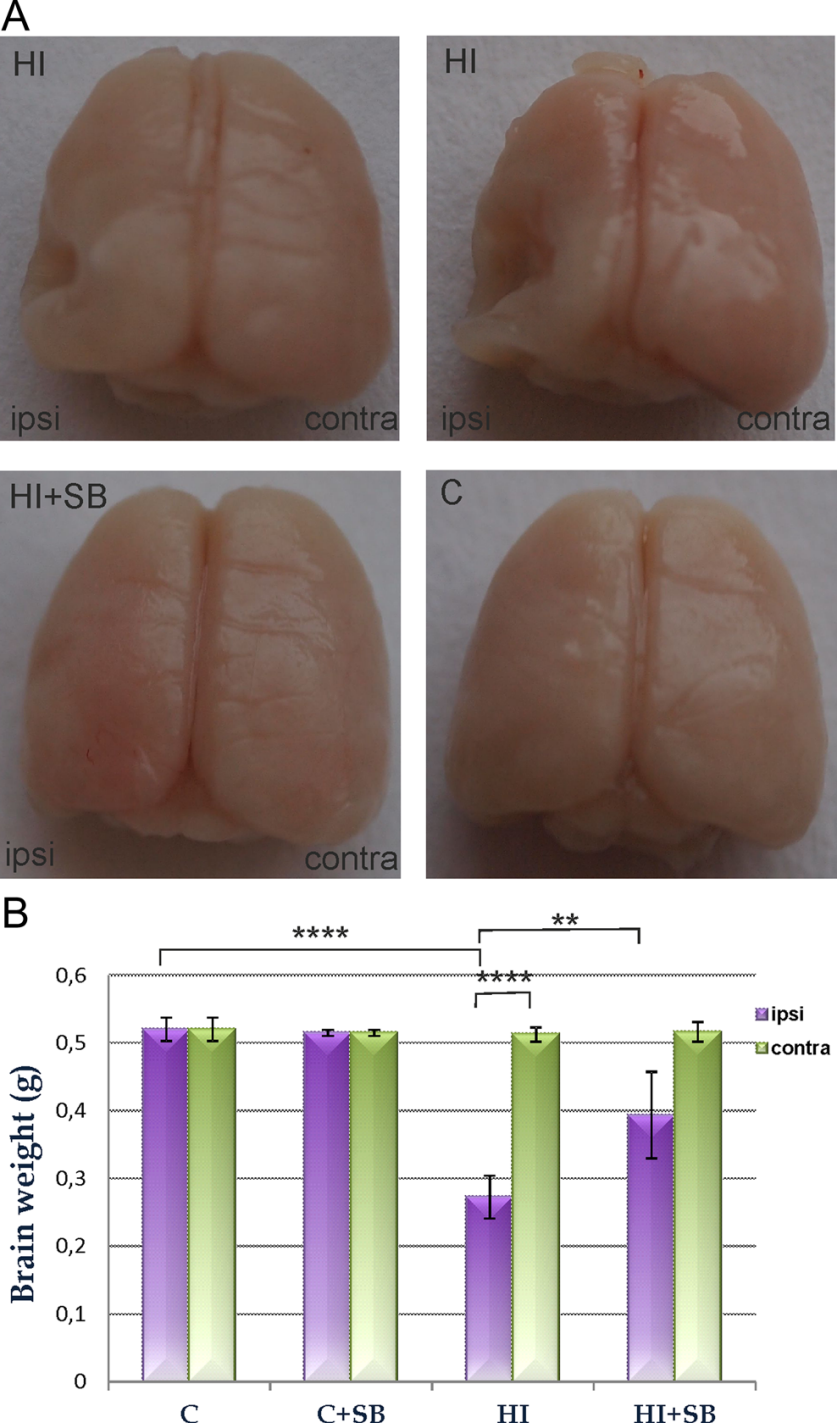
Sodium butyrate (SB) reduces brain damage after neonatal HI. Neonatal HI was induced at postnatal day 7. SB was administered directly after the onset of HI and for 5 consecutive days. Brain damage was evaluated 14 days after HI. The degree of damage was demonstrated as the weight deficit of the ipsilateral (injured hemisphere) compared to the contralateral one. **a** Dorsal view of rat brains at P21. Note the brain asymmetry and atrophy in ipsilateral (*left*) hemisphere after HI (*upper panel*). Treatment with SB after HI (HI+SB) prevents the atrophy; however, a small degree of asymmetry is still visible (*lower left image*). Abbreviations: *C* control, *HI* hypoxia ischemia. **b** Weight (g) of the ipsilateral and contralateral hemispheres in rats with or without SB treatment. The values are mean ± SD of 5 animals in each experimental group. One-way ANOVA and Bonferroni test **p < 0.01 and ****p < 0.0001 indicate statistically significant differences. Abbreviations: *C* control, *ipsi* ipsilateral, *contra* contralateral

All pups exposed to HI were lesioned in the left (ligated) hemisphere. Dorsal view of the rat brain 2 weeks after the insult presents damage (atrophy) of the ipsilateral hemisphere and brain asymmetry. It should be noted that this model of HI leads to different degree of damage reflecting individual response to the insult (please compare Fig. 1a upper panel). After SB treatment, none of the ligated hemispheres showed atrophy. However, in a few cases, low degree of brain asymmetry could still be visible (Fig. 1a lower panel left image).

In order to evaluate the effect of sodium butyrate on brain damage caused by neonatal hypoxia-ischemia, we compared the wet weight of ipsilateral hemispheres to the contralateral ones. Two weeks after hypoxia ischemia, the mean weight deficit of the ipsilateral (injured) hemisphere amounted 49.81 %, and it decreased to 27.05 % after SB treatment (Fig. 1b). In the same conditions, the weight of the intact contralateral side was not different from the respective sham control.

### The Effect of SB on the Acetylation of Histone 3

**Fig. 2:**
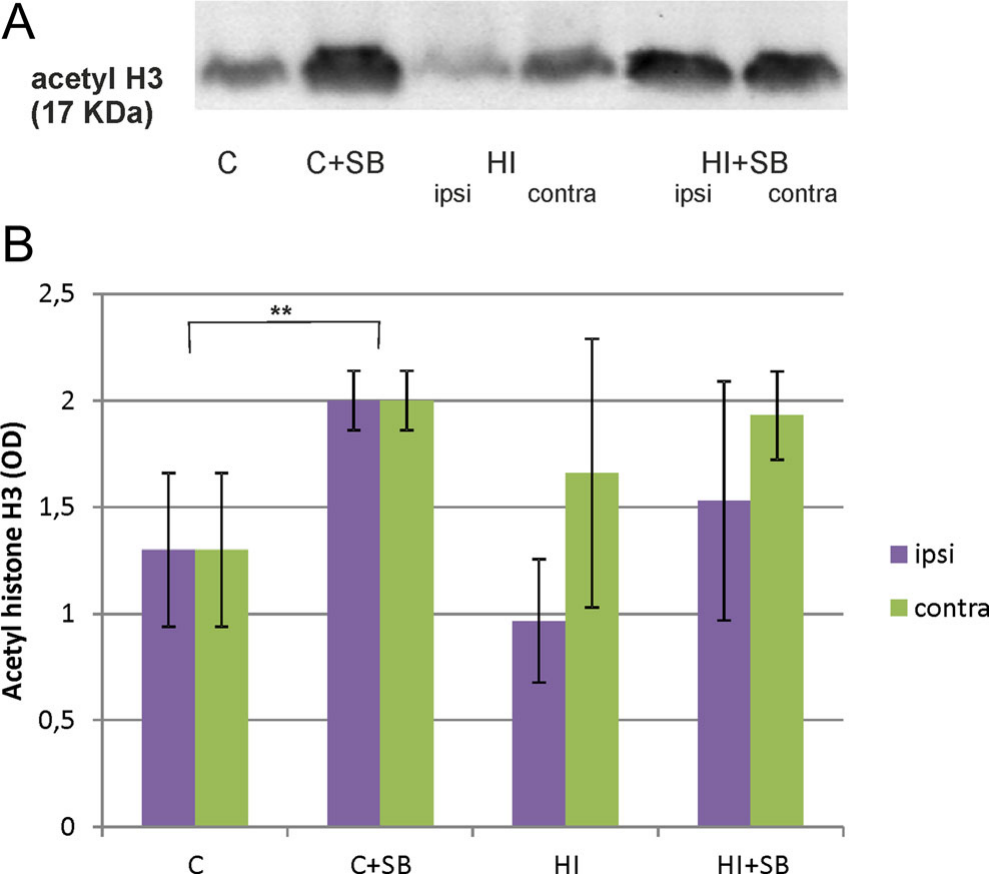
Sodium butyrate increases acetylation of histone H3 only in control animals. **a** Representative immunoblot of AcH3 showing the effect of SB in control animals and 72 h after HI, analyzed in four experimental groups: vehicle control (*C*), SB-treated control (C+SB), vehicle-treated hypoxia-ischemia (HI), SB-treated hypoxia-ischemia (HI+SB). The intensity of each band was quantified by LKB Ultrascan XL, Program Gel Scan, and normalized in relation to beta-actin. **b** Statistical analysis of densitometric data from indicated experimental groups. The values are mean ± SD from 5 animals that were assessed in 3 independent experiments. One-way ANOVA and Bonferroni test **p < 0.01, control+SB vs. vehicle control. Abbreviations: *C* control, *ipsi* ipsilateral, *contra* contralateral

At first we measured the level of acetylated histone 3 (AcH3) in rat brains after the application of 1, 3, or 5 doses of sodium butyrate. A noticeable effect of SB was observed only after 3 days of treatment, which corresponds to postnatal day 10 (72 h of recovery after HI). Figure 2 shows respective representative immunoblots probed with antibody specific to acetylated histone H3 together with the densitometric analysis. As depicted, the injection of SB resulted in significant increase of AcH3 immunoreactivity by 54 % over vehicle control, only in sham-operated animals. In contrast, no statistically significant changes in AcH3 level were noted in the investigated brains after HI insult.

### Time Course of Cell Proliferation in the Brain Hippocampus

**Fig. 3:**
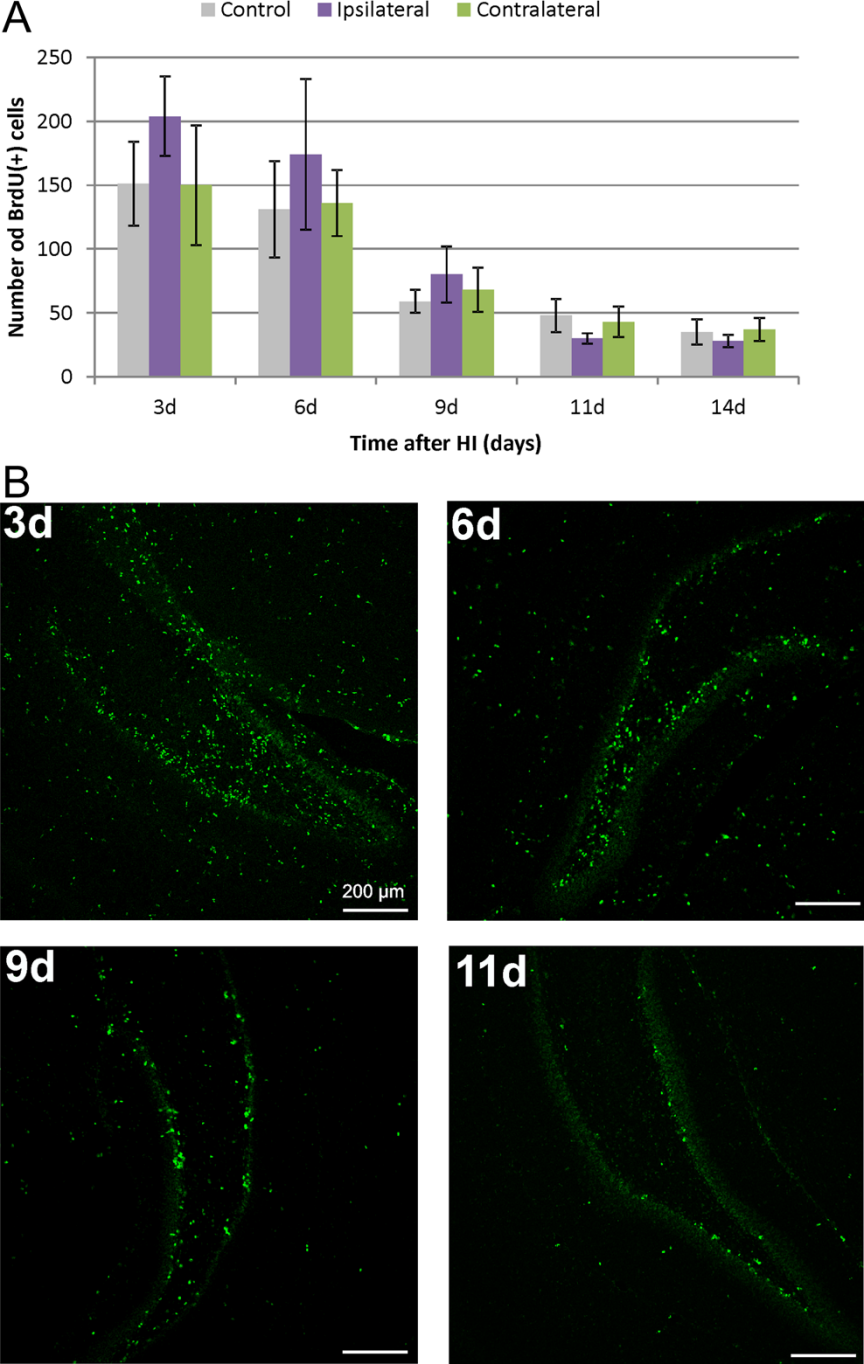
Time course of cell proliferation in DG of the rat hippocampus Animals received a single dose of BrdU (50 mg/kg) 24 h prior to sacrifice at 3, 6, 9, 11, 14 days after HI and the brains were processed for BrdU immunohistochemistry. **a** The number of BrdU-labeled nuclei within the DG area in sham-operated control animals and at different times after ischemia in both sides: injured ipsilateral and non-injured contralateral. The highest number of proliferating cells was detected at 3–6 days after the injury, thereafter the density of BrdU-positive cells markedly decreased. The values are mean ± SD of 5 animals per group and time point. One-way ANOVA and Bonferroni test did not indicate significant differences in BrdU labeling between control (*gray bars*), ipsilateral (*purple bars*), and contralateral hippocampi (*green bars*) in investigated time points. **b** Newly divided cells in the HI-injured ipsilateral DG of the rat hippocampus. Confocal photomicrographs show immunohistochemical reactions at 3, 6, 9, and 11 days after HI. While at 3 days of recovery the greatest number of BrdU-positive cells is seen in the hilus, with the prolongation of recovery time, their number becomes particularly pronounced in the neurogenic SGZ of the hippocampus. *Scale bar* 200 μm

The time course of cell proliferation in the rat hippocampus was studied at specific time points after hypoxic-ischemic injury (3, 6, 9, 11, and 14 days after HI). For this purpose, animals received a single dose of BrdU 24 h prior to sacrifice. The number of newly generated cells during 24 h was determined in the entire DG region of the hippocampus by monitoring the incorporation and subsequent immunohistochemical detection of BrdU. BrdU-labeled cells were detected in all evaluated animals. As evidenced by the analysis of hippocampal sections, there was no difference in the number of proliferating cells (BrdU+) between injured and control, sham-operated animals. The highest density of BrdU incorporation was detected between 3 and 6 days of recovery. Thereafter, cell proliferation decreased markedly in all experimental groups, almost sevenfold, as compared with the primary time point, indicating lowering of the dynamic of stem/progenitor cell proliferation (Fig. 3a).

Interestingly, the exposure to SB did not affect the number of BrdU-positive cells in DG–neither in ipsi- nor in contralateral side.

The distribution of BrdU-labeled cells throughout the brain varies between the topographical areas. At 3 days of recovery, a great number of dividing cells was seen in the hilus area. With the prolongation of recovery to 9 and 11 days, BrdU+ cells are located almost exclusively in the neurogenic SGZ of the DG (Fig. 3b).

### Phenotypic Characterization of Proliferating Cells after Neonatal Hypoxic/Ischemia

To further characterize the fate of newly arisen cells after SB treatment, brain tissue sections from sham and HI rats were double-stained for BrdU and different neural antigens–DCX (for neuroblasts), calbindin (for mature granule neurons), NG2 (detected in oligodendrocyte progenitor cells), O4 (for immature, non-myelinating oligodendrocytes), and MBP (for mature myelinating oligodendrocytes). For the purpose of these studies, animals received multiple BrdU injections on days 4–6 after hypoxia-ischemia and were sacrificed at 14 or 28 days of recovery. At first we investigated whether the treatment with SB influenced cell proliferation in the hippocampus. A striking proliferation of the precursor population was seen at the investigated periods of recovery in the DG of control and HI-injured rats, with the most pronounced expansion of BrdU-positive cells at postnatal day 21 (which is equivalent to 14 days after the insult). No significant differences were seen in the number of cells incorporating BrdU when the hypoxic-ischemic DG was compared with the control. Also, the treatment with SB did not change the pattern of proliferation response.

**Fig. 4:**
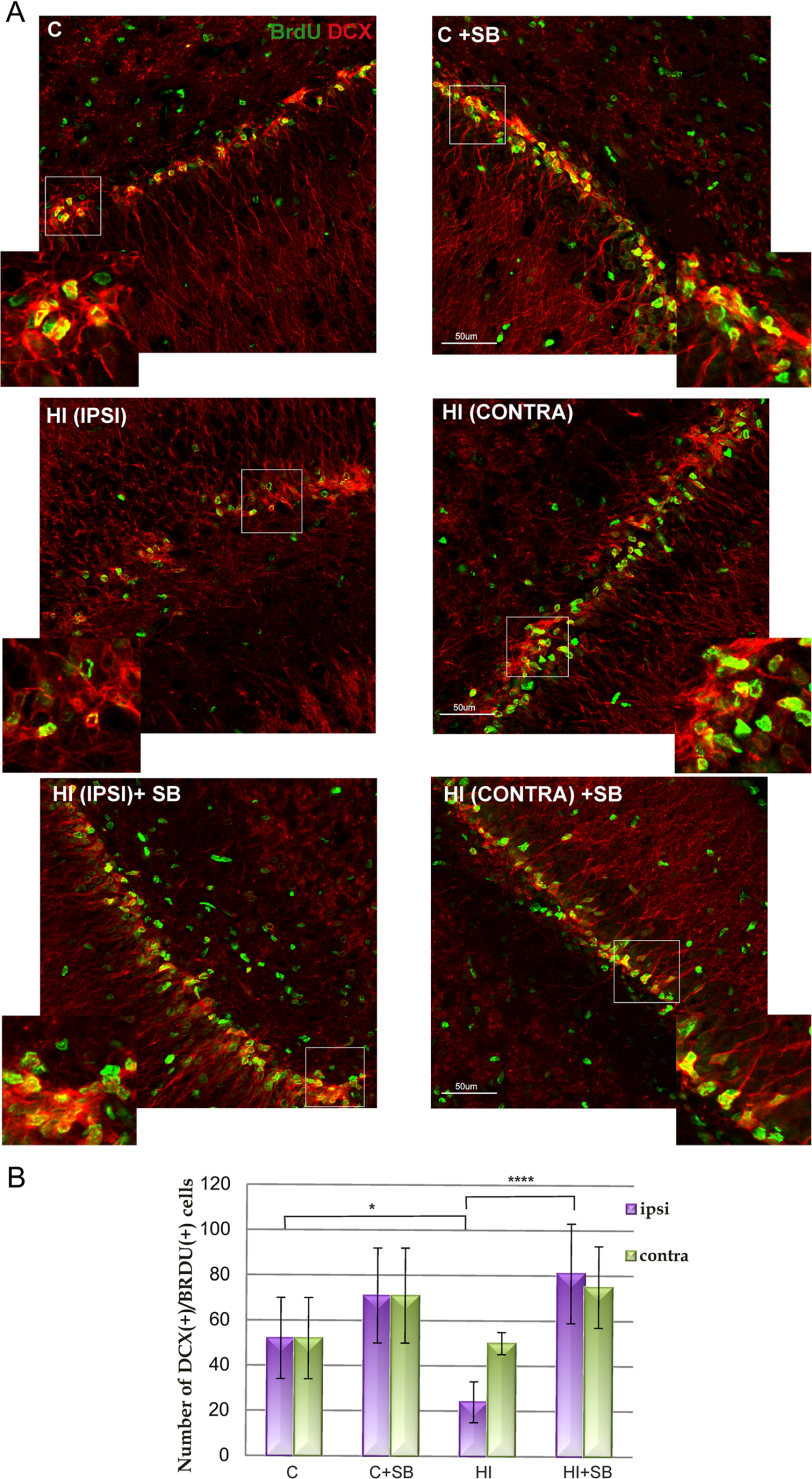
Sodium butyrate promotes generation of new neuroblasts in the DG after HI. Neonatal HI was induced at postnatal day 7. SB was administered directly after the onset of HI and for 5 consecutive days. BrdU was administered twice daily for 3 consecutive days starting 4 days after the onset of HI. Brain sections from control animals and from animals 14 days after HI were double-labeled with anti-BrdU (*green*) and anti-DCX (*red*) antibodies. **a** Confocal photomicrographs show double-labeled cells in DG of control (*C*) and HI animals 14 days after the insult, with or without SB treatment. *Inserts* represent magnification. Note that HI led to a marked decrease of BrdU/DCX-positive cells in the injured, ipsilateral side, and the density of new neuroblasts clearly increased after SB treatment. **b** Number of BrdU/DCX-positive cells quantified in the DG area (0.36 mm2) in sham-operated control animals and 14 days after HI in the injured ipsilateral as well as in the non-injured contralateral side. The values are mean ± SD of 5 animals per experimental group. One-way ANOVA and Bonferroni test indicate significant differences in double labeling between investigated groups, *p < 0.05 ipsilateral HI vs. control and ****p < 0.0001 ipsilateral HI vs. ipsilateral HI+SB. Abbreviations: *IPSI* ipsilateral, *CONTRA* contralateral

Double fluorescent studies revealed numerous BrdU-positive nuclei in the neurogenic subgranular zone of DG closely associated with neuronally committed precursors and/or immature neurons (neuroblasts) expressing a microtubule associated protein-DCX. DCX-positive cells were extensively distributed in the DG of sham-operated control animals, as well as of the group treated with SB (Fig. 3a upper panel and Fig. 3b). As shown by labeling and subsequent counts, the number of BrdU/DCX+ cells was significantly decreased (by about 50 %) in the hypoxic-ischemic side at 14 days of recovery compared with controls (mean counts 24 vs. 50 BrdU/DCX-positive cells, respectively; *p* < 0.05). In contrast, the number of cells in the side contralateral to ligation, although also exposed to hypoxia, remained close to the controls. It clearly appears that the administration of sodium butyrate substantially increased BrdU/DCX-positive cells in HI side to the value presented in sham (Fig. 4a left images in the middle and lower panel and Fig. 4b). We also observed that the number of neuroblasts slightly increased within the DG of the hypoxic side; however, the changes were not significant as compared to the vehicle-treated animals (Fig. 4b).

**Fig. 5:**
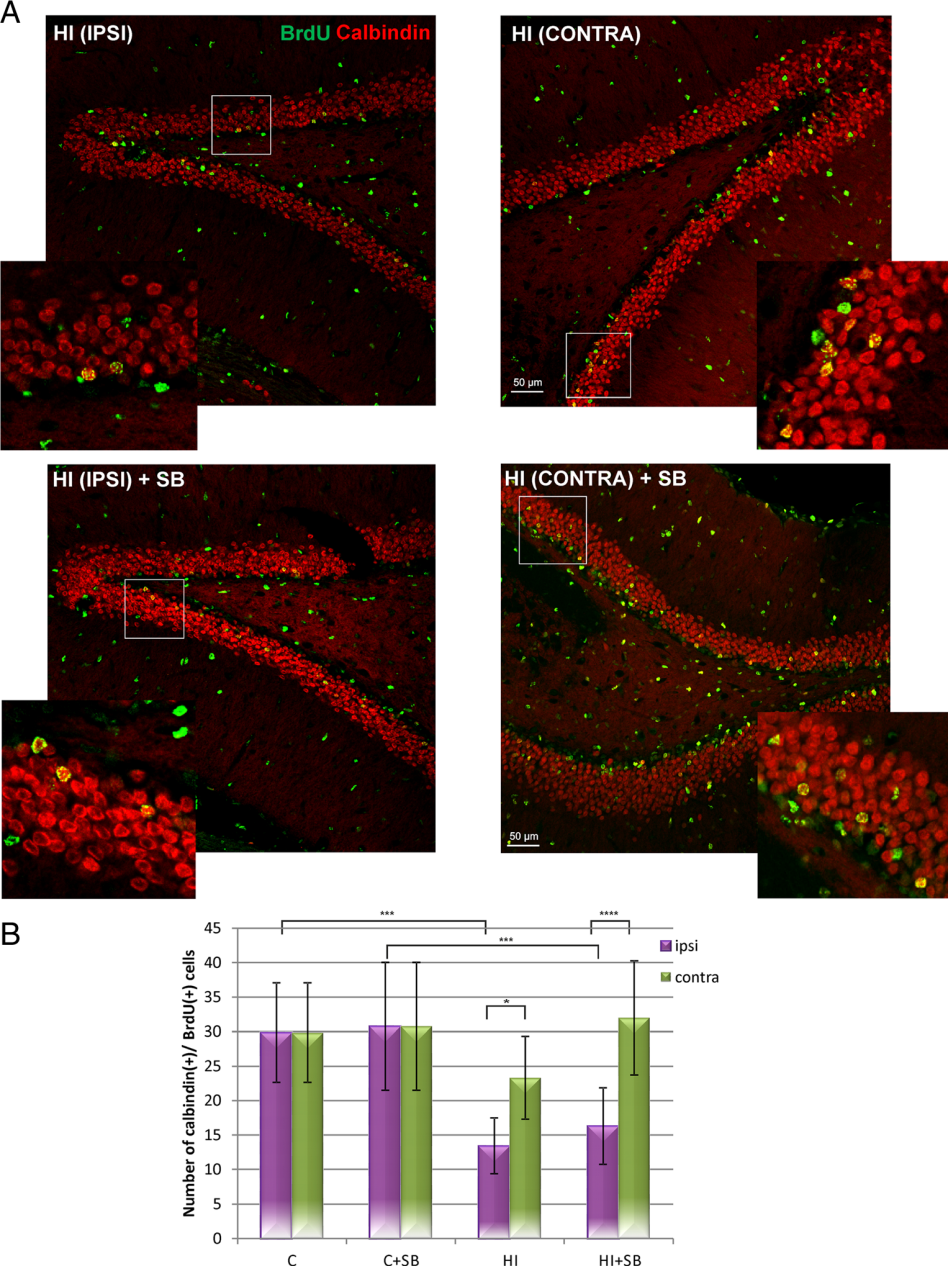
Sodium butyrate does not increase the number of newly generated granule neurons in the DG after HI. Neonatal HI was induced at postnatal day 7. SB was administered directly after the onset of HI and for 5 consecutive days. BrdU was administered twice daily for 3 consecutive days starting 4 days after the onset of HI. Brain sections from control animals and from animals 28 days after HI were double-labeled with anti-BrdU (*red*) antibody and the granule cell marker-calbindin (*green*). **a** Confocal photomicrographs show double-labeled cells in the ipsilateral (injured) and contralateral side of DG 28 days after HI with or without SB treatment. *Inserts* represent magnification. Photomicrographs are representative of observations made from 5 animals per experimental group. *Scale bar* 50 μm. **b** Number of BrdU/calbindin-labeled cells quantified in the DG area (0.36 mm2). Values represent mean ± SD of 5 animals per time point. One-way ANOVA and Bonferroni test indicate significant differences in double-labeled cells between investigated groups, *p < 0.05 ipsilateral HI vs. contralateral HI, ***p < 0.001, ipsilateral HI vs. control, ****p < 0.0001, ipsilateral HI+SB vs. contralateral HI+SB. Abbreviations: *IPSI* ipsilateral, *CONTRA* contralateral

To check whether neuroblasts localized in the subgranular zone of DG differentiate to mature neurons, we performed double labeling assay using BrdU with calbindin—a marker of granule neuronal cells. Quantified results are shown in Fig. 5b. It clearly appears that 28 days after hypoxic-ischemic injury, there is a significant decrease (more than 50 %) in double-stained BrdU/calbindin+mature granule neurons in the DG region of the ipsilateral (hypoxic-ischemic) side compared with controls (*p* < 0.001), without significant effect noticed contralaterally. In contrast to the beneficial effect of SB on the number of neuroblasts in the ipsilateral DG, in the same applied conditions, the inhibitor did not increase the amount of newly generated granule neurons, which remained persistently lower than control.

**Fig. 6:**
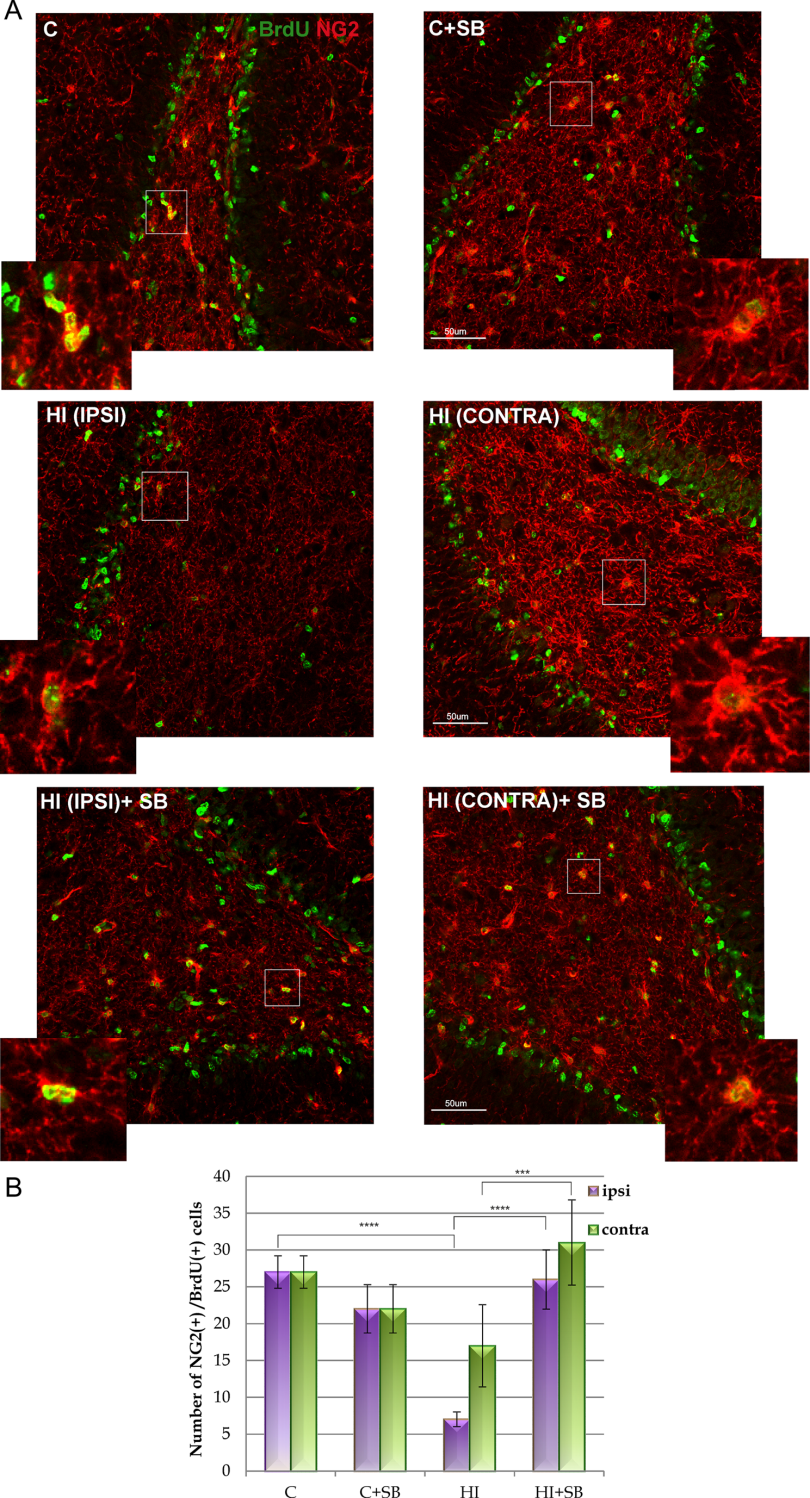
Sodium butyrate stimulates oligodendrocyte precursor cell proliferation in DG of the rat hippocampus after HI. Neonatal HI was induced at postnatal day 7. SB was administered directly after the onset of HI and for 5 consecutive days. BrdU was administered twice daily for 3 consecutive days starting 4 days after the onset of HI. Brain sections from control animals and from animals 14 days after HI were double-labeled with anti-BrdU (*green*) antibody and the oligodendrocyte precursor cell marker–NG2 (*red*). **a** Confocal photomicrographs of control and HI ipsilateral and contralateral side of DG with or without SB treatment. Note the decreased number of double-labeled cells (BrdU/NG2-positive) in the ipsilateral side as compared to the control. The density of new oligodendrocyte progenitors increases after SB treatment in both hemispheres-ipsi- and contralateral. *Inserts* represent magnification. Photomicrographs are representative of observations made from 5 animals per experimental group. *Scale bar* 50 μm. **b** Graph shows the number of BrdU/NG2-labeled cells quantified in the DG area (0.36 mm^2^). Values represent mean ± SD of 5 animals per experimental group. One-way ANOVA and Bonferroni test indicate significant differences in double-labeled cells between investigated groups, ***p < 0.001, contralateral HI vs. contralateral HI+SB, ****p < 0.0001, ipsilateral HI vs. control, and ipsilateral HI vs. ipsilateral HI+SB. Abbreviations: *C* control, *IPSI* ipsilateral, *CONTRA* contralateral

In order to evaluate the differentiation of newly produced cells to oligodendroglial phenotype, we carried out double fluorescent studies for BrdU and specific markers. To address the question whether SB stimulates oligodendrogenesis within the DG area, we analyzed the number of proliferating oligodendrocyte precursor cells (OPCs) as well as more mature oligodendrocytes in the sham and hypoxic-ischemic animals after 2 and 4 weeks. For this purpose, we used antibodies specific for cells being at a different developmental stage. In control we observed a number of BrdU-labeled cells co-stained with NG2, commonly used for the identification of OPCs. As is shown in Fig. 6, 2 weeks after the hypoxic-ischemic insult, we noted a marked decrease in new OPCs (by about 75 % compared to sham control) ipsilaterally (*p* < 0.0001); while the number of these cells in the contralateral hypoxic side did not present statistical difference from the control. Exposure to SB resulted in a significant raise in the number of new progenitors in the ipsilateral (*p* < 0.001) as well as in the contralateral (*p* < 0.01) side. The restoration of the lost OPCs to the level of sham control and maintained further at 4 weeks of recovery could be due, at least in part, to the neuroprotective effect of this agent.

**Fig. 7:**
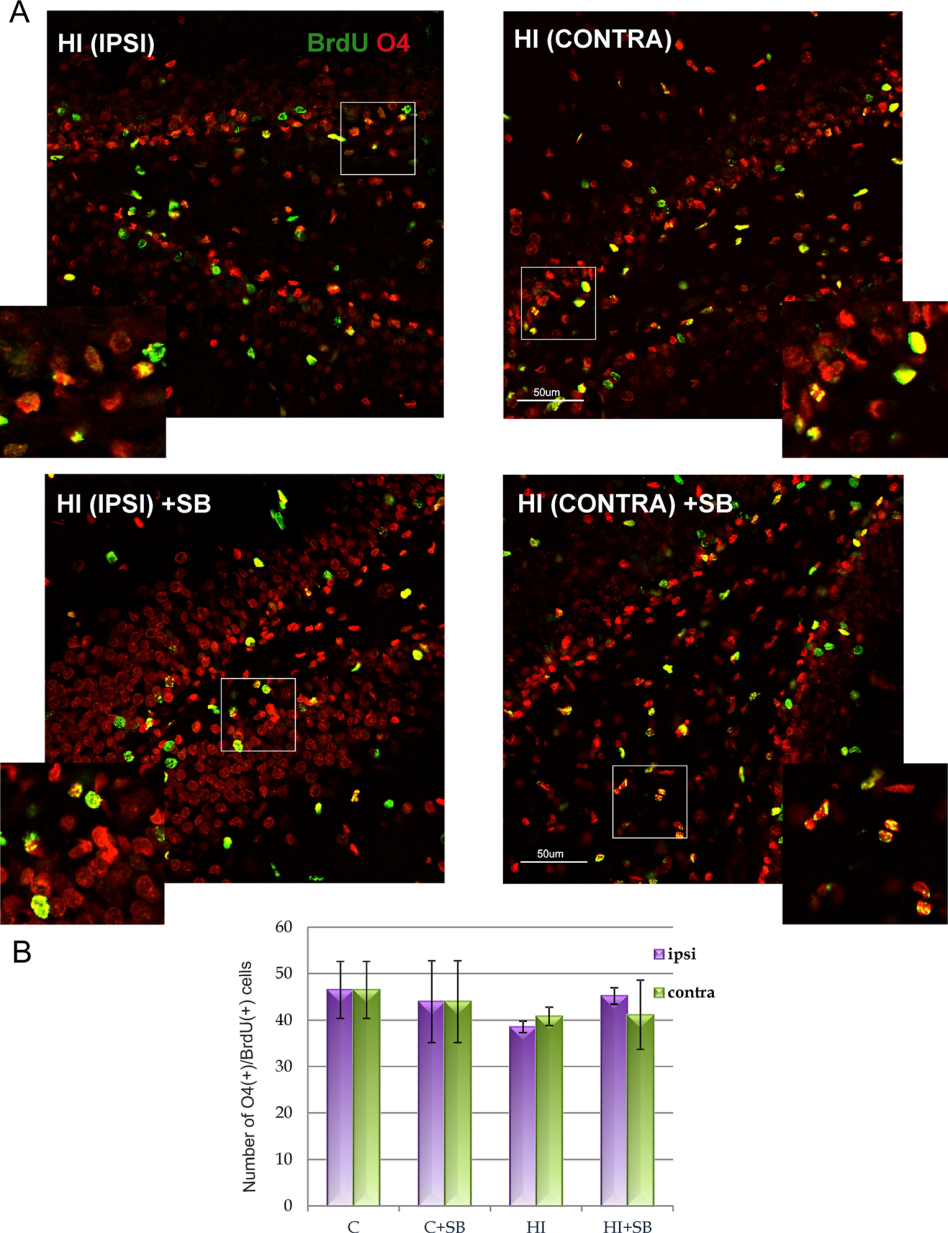
SB does not influence the number of oligodendrocytes expressing O4 in DG of the rat hippocampus after HI. Neonatal HI was induced at postnatal day 7. SB was administered directly after the onset of HI and for 5 consecutive days. BrdU was administered twice daily for 3 consecutive days starting 4 days after the onset of HI. Brain sections from control animals and from animals 28 days after HI were double-labeled with anti-BrdU antibody (green) and the non-myelinating oligodendrocyte marker–O4 (*red*). **a** Confocal photomicrographs show double-labeled cells in the ipsilateral (injured) and contralateral side of DG 28 days after HI with or without SB treatment. *Inserts* represent magnification. Note the similar density of BrdU/O4-positive cells in indicated sections. Photomicrographs are representative of observations made from 5 animals per experimental group. *Scale bar* 50 μm. **b** Number of BrdU/O4-labeled cells quantified in the DG area (0.36 mm^2^). Values represent mean ± SD of 5 animals per time point. One-way ANOVA and Bonferroni test did not indicate significant differences in the number of double-labeled cells between the investigated groups. Abbreviations: *C* control, *IPSI* ipsilateral, *CONTRA* contralateral

Moreover, it was found that at the same time point (4 weeks), the density of cells double-stained with BrdU/O4 (marker of the later-stage progenitor cells) in injured animals did not differ from control group, as well as no differences were noted in the density of cells that express MBP (marker of mature oligodendrocytes). In addition, there was no visible effect of SB either on the number of BrdU/O4 (Fig. 7) or BrdU/MBP cells (data not shown).

**Fig. 8:**
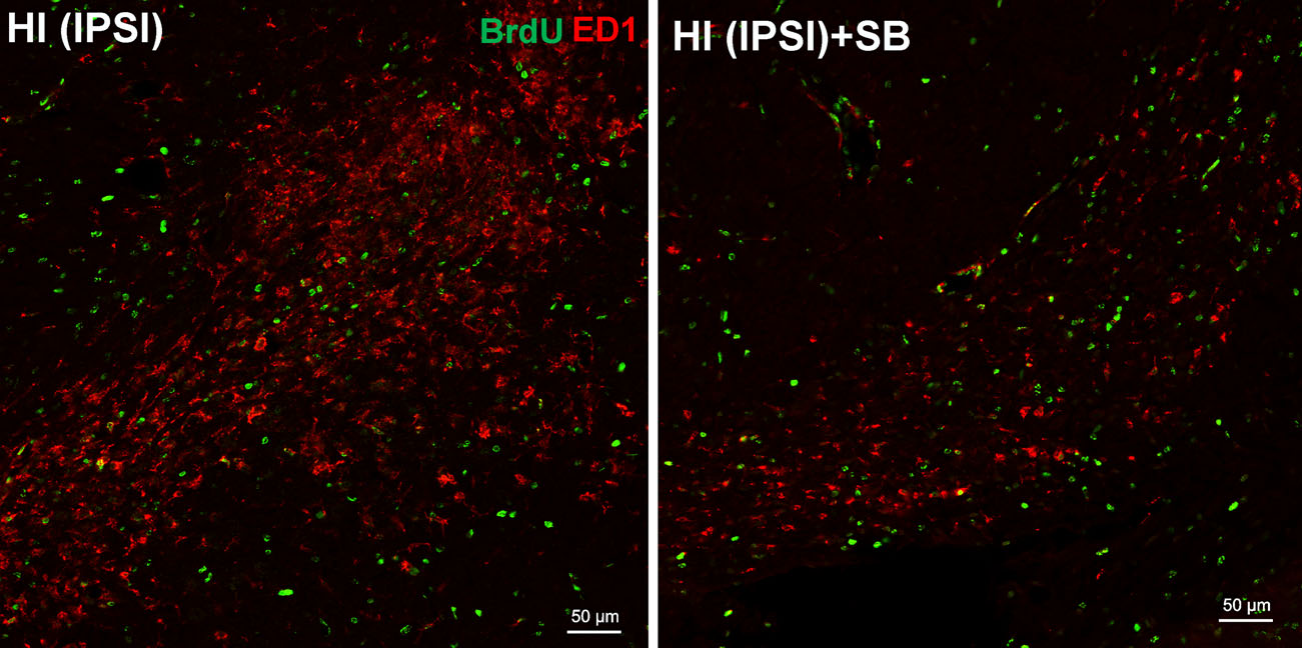
Sodium butyrate reduces microglial cell number in the rat ipsilateral hemisphere after HI. Neonatal HI was induced at postnatal day 7. SB was administered directly after the onset of HI and for 5 consecutive days. BrdU was administered twice daily for 3 consecutive days starting 4 days after the onset of HI. Brain sections from an SB-treated as well as untreated ipsilateral rat hemispheres were double-labeled with anti-BrdU (*green*) and ED1 (marker for microglia and macrophages) antibody 14 days after HI. Confocal photomicrographs of ipsilateral hemispheres show a large number of double-stained cells (BrdU/ED1-positive) after HI and marked reduction after SB treatment. Photomicrographs are representative of observations made from 5 animals per experimental group. *Scale bar* 50 μm. Abbreviations: *IPSI* ipsilateral

Subsequently, based on several studies suggesting a strong association of HI with inflammation, we examined the density of microglial cells labeled for BrdU. As shown in Fig. 8, the ipsilateral hemisphere exhibited a large number of microglial cells assessed by double staining BrdU/ED1 at 14 days after HI. SB treatment significantly suppressed cells expressing the ED1 marker in the ligated hemisphere. It is important to point out that these cells were not seen either in the contralateral side or in age-matched sham-operated animals.

### Contribution of Endogenous BDNF to SB Induced Neurogenesis

**Fig. 9:**
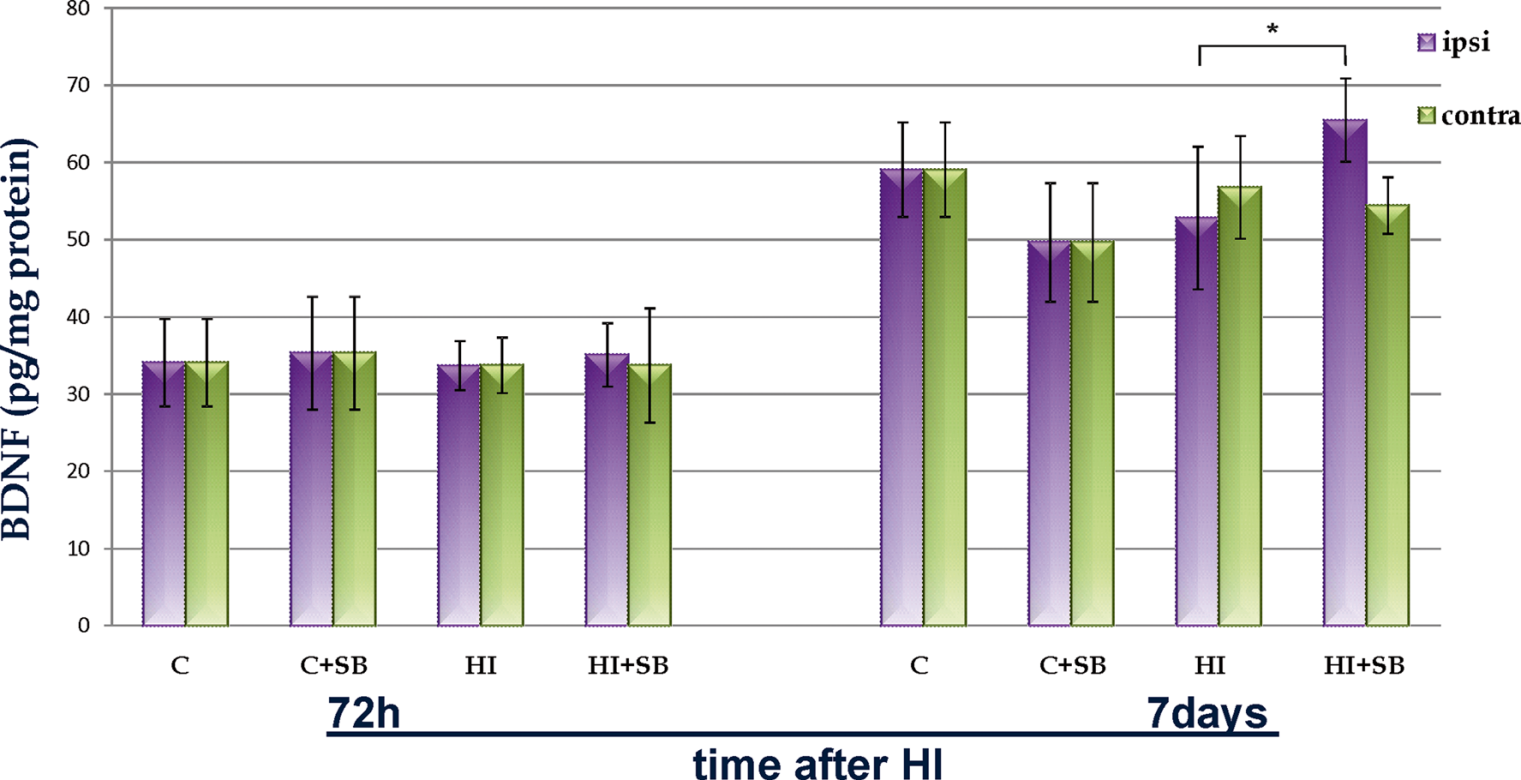
Sodium butyrate increases the level of BDNF in the ipsilateral hemisphere after HI. Graph represents the BDNF level in cerebral hemispheres (ipsi- and contralateral) in four investigated groups: vehicle-treated control (C), SB-treated control (C+SB), vehicle treated hypoxia-ischemia (HI), and SB-treated hypoxia-ischemia (HI+SB). The values are mean ± SD from 5 animals per group that were assessed in two independent experiments. The level of BDNF was significantly higher 7 days after HI as compared to 3 days of recovery. One-way ANOVA and Bonferroni test, *p < 0.05, indicate statistically significant differences between ipsilateral hemispheres from rats receiving SB (HI+SB) vs. vehicle (HI)

According to the data indicating involvement of BDNF in neurogenesis, maturation, and survival of newborn cells after ischemic insult, we assessed whether the SB-induced generation of new cells correlates with the level of this neurotrophin. BDNF was examined in hippocampi and in cerebral hemispheres 3 and 7 days after hypoxia ischemia. We found that in each experimental condition, the level of BDNF was significantly higher at postnatal day 14 (corresponding to 7 days of recovery) as compared to P10 (3 days of recovery) by about 36 % in average. After hypoxic-ischemic injury, the amount of BDNF remained close to the control value in both investigated time points. The animal treatment with SB led to an increase of BDNF level in the ipsilateral side at 7 days after HI (*p* < 0.05; Fig. 9). The same direction of changes was observed in the hippocampus; however, all described responses were more subtle and did not show significance.

### Behavioral Testing

#### Open Field

**Fig. 10:**
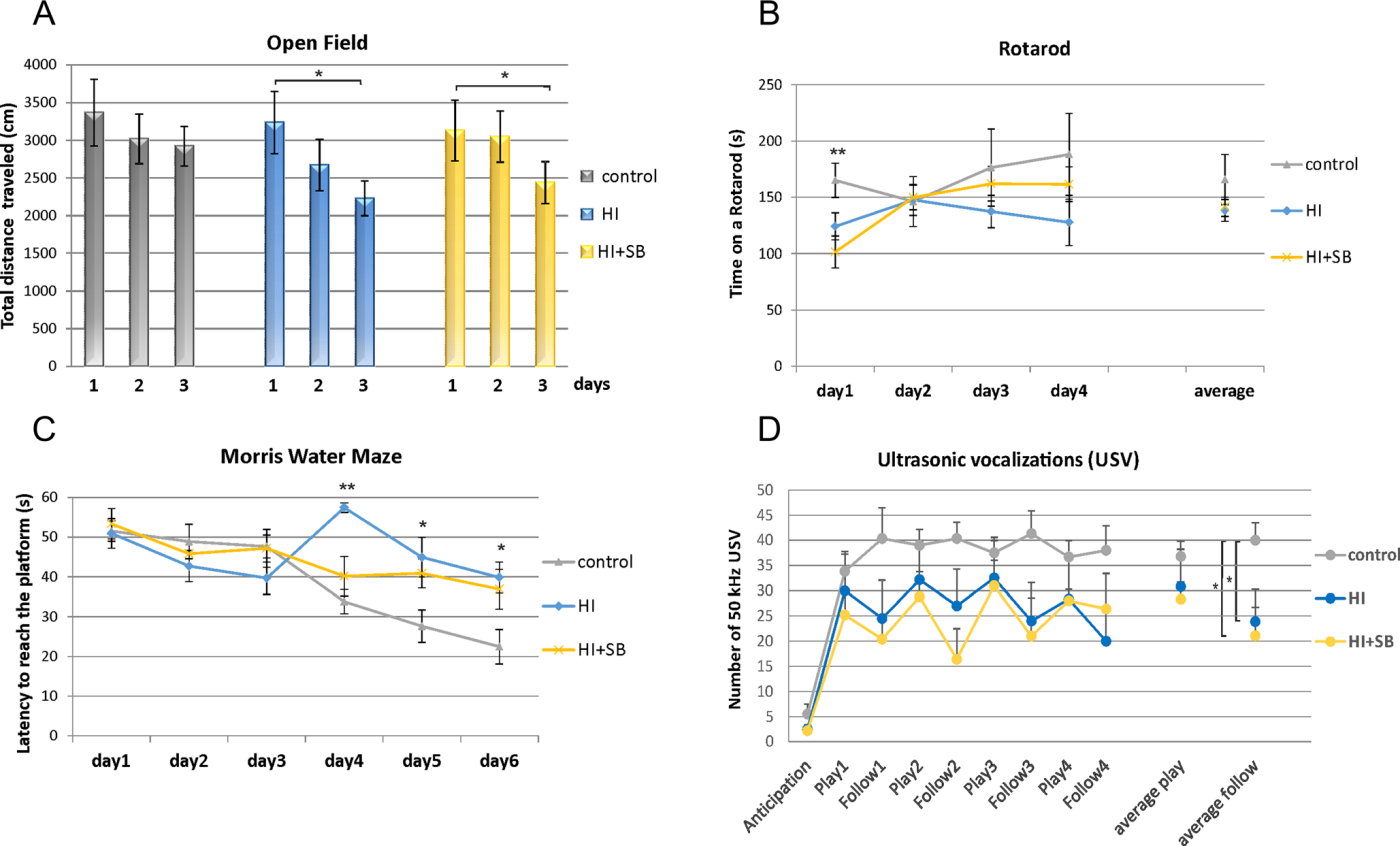
SB treatment does not affect behavioral outcome after HI. Animal behavior was monitored at P33–79 in three investigated groups: control (*C*), hypoxia-ischemia (*HI*), and hypoxia-ischemia treated with SB (*HI+SB*). *Schemes* represent the data of indicated tests. Open field (**a**) and RotaRod (**b**) test results show a lack of difference between investigated groups (control, HI, HI-treated with SB). Morris water maze (MWM) (**c**) and ultrasonic vocalizations (USV) recording (**d**) results illustrate behavioral deficiency in rats after HI with some tendency for improvement after SB treatment in MWM. The results are presented as mean ± SEM. One-way ANOVA and Duncan test *p < 0.05, **p < 0.01

On P33–34 the open field test was performed to assess the animals’ response to a novel environment, their activity, and locomotion abilities. There was an effect of the day *F*(2,62) = 7.3, *p* < 0.01, reflecting shortening of the distance traveled in consecutive days, but no effect of the group *F*(2,31) = 0.5, *p* = 0.61, and no group × day effect *F*(4,62) = 1.0, *p* = 0.42. Day differences were confirmed by post hoc analysis in HI group (day 1 vs. day 3, *p* < 0.05) and in HI+SB group (day 1 vs. day 3, *p* < 0.05) (Fig. 10a). The results of velocity paralleled these results. There were no other differences for duration and frequency in OF zones. Results of this experiment reflect that the administration of SB does not influence the response of animals to novelty or their locomotor skills.

#### Rotarod

On P39–40 the rotarod test was carried out to assess coordination, balance and gross motor functions. There was only an effect of the day, *F*(3,60) = 3.4, *p* < 0.05, reflecting general tendency for the animals to stay on the rod longer from day to day, with no group effect *F*(2,20) = 2.1, *p* = 0.15. However, a direct comparison (one-way ANOVA) of the first day results between sham control (165.1 ± 15.2 N) and HI animals (124.1 ± 12.1 N) (*F*(1,14) = 4.1, *p* = 0.06) and between control and HI+SB rats (101.4 ± 14.0 N) (*F*(1,19) = 9.3, *p* < 0.01) suggest that the latency to fall was reduced in HI rats compared to age-matched controls (Fig. 10b).

#### Grip Test

To quantify the impact of HI and SB administration on the muscular strength of animals, we used the grip test starting from day P46–47. There was no group (*F*(2,32) = 1.5, *p* = 0.24), and no day (*F*(1,32) = 0.1, *p* = 0.74) effect, illustrating that neither HI nor SB affected the grip strength of forelimbs.

#### Morris Water Maze

Spatial learning and memory were assessed in animals starting from P62 using the Morris water maze test. We noticed longer latencies to reach the platform in hypoxia-ischemia rats when compared to control animals, while SB treatment had no major influence. After several days of learning, all animals showed shorter latencies in reaching the platform, with a strong effect of the day *F*(5160) = 9.3, *p* < 0.001. There was also a general effect of the group *F*(2,32) = 3.8, *p* < 0.05 as well as a group × day effect *F*(10,160) = 3.7, *p* < 0.001. Post hoc analysis showed a decrease in latency to reach the platform (Fig. 10c) in control rats (*p* < 0.001) as well as in HI+SB group (*p* < 0.01) and almost significant effect in HI rats (*p* = 0.08). Also, toward the end of the training (days 4–6), there was a difference between control and HI rats on day 4 (*p* < 0.01), day 5 (*p* < 0.05), and day 6 (*p* < 0.05), whereas there was no significant difference between control and HI+SB rats during these days (*p* > 0.05, Duncan). However, the results of HI+SB animals closely paralleled these of HI rats toward the end of the training. Thus, results obtained from this test suggest that HI animals have a more impaired memory performance than those with SB administration when compared to control animals.

#### Ultrasonic Vocalizations (USV)

The evaluation of social and emotional behaviors was done by measuring the rates of 50 kHz USV. HI and HI+SB animals displayed less USV then control animals, especially when following the experimenter’s hand. There was no significant effect of SB administration observed, i.e., no difference between HI and HI+SB groups. However, as can be observed in Fig. 10d, the number of USV emitted by control animals was higher than in case of HI and HI+SB rats. This effect was especially visible during follow sessions. When average numbers of USV from all follow sessions were analyzed (ANOVA), there was the effect of group *F*(2,14) = 3.8, *p* < 0.05; with differences between control (40.0 ± 3.5 USV) vs. HI (23.9 ± 6.4) animals (*p* < 0.05) as well as control vs. HI+SB rats (21.1 ± 5.6) (*p* < 0.05, Duncan). While there was no group effect for average USV emitted during play sessions, *F*(2,14) = 0.7; *p* = 0.51; control rats, 36.8 ± 3.0; HI rats, 30.8 ± 7.4; HI+SB rats, 28.3 ± 3.4 USV). Results obtained in this task suggest that HI affected USV emissions at anatomical and/or behavioral levels.

## Discussion

The present study showed that treatment with histone deacetylase inhibitor-sodium butyrate after HI provides neuroprotection. The neuroprotective effect of HDACi was connected with a reduction of brain damage when measured at 14 days after the onset of hypoxia-ischemia, as well as increased neurogenesis. The striking features of our results were that neuroprotective/neurogenic effects of SB observed in our study were associated with expanded population of neuroblasts detected by staining with BrdU/DCX, expansion of oligodendrocyte precursor cells (BrdU/NG2-positive) as well as inhibition of HI-induced inflammation. However, in contrast to our expectation, we did not observe significant improvement of the behavioral impairments seen in MWM and USV, which resulted from HI.

Neonatal hypoxia-ischemia sets in motion a series of pathophysiological processes that result ultimately in the massive loss of neurons and severe neurological deficits, despite the enhanced neurogenic response subsequent to neonatal brain injury. It is of note that many experimental studies have shown an increased number of proliferating neural progenitor cells residing in the active SVZ. Furthermore, these give rise to neuroblasts that migrate into the ischemia-damaged structure. However, in spite of these interesting and important findings, there remains much doubt regarding the regenerative capacity because the expected long-lasting effect on neuron numbers was not noticed [6, 46, 47]. A few papers evaluating the proliferative capacity in the second neurogenic area-hippocampal SGZ, reported inconsistent data: either increased [46] or reduced [7, 48] total counts of new cells. These obvious discrepancies could be explained by differences in experimental conditions including, among others, BrdU injection protocols, which undoubtedly contribute to the variability in the number of BrdU-positive cells among the animals. During the course of our study, we selected a BrdU administration protocol (4–6 days after lesioning) on the basis of our experimental results as well as on the results of adult rodent stroke studies [49]. We anticipated that this paradigm would be optimal for labeling newly generated cells. Quantitative analysis of BrdU labeling in tissue samples gained 2 and 4 weeks after the injury shows that the population of proliferating cells in the ipsilateral as well as in contralateral DG is close to age-matched sham-operated animals. This observation is consistent with data reported previously by Qiu et al. [50] in mice subjected to HI. Thus, it may be supposed that the immature hippocampus is already working at the top of its proliferative capacity in this stage of development. It is also probable that the pool of progenitors might be preferentially protected against ischemic depletion. The unchanged intensity of proliferation found in this study remains in striking contrast with a marked suppression of the population of new neuroblasts (BrdU/DCX) as well as newly matured granule neurons (BrdU/calbindin-positive) in the HI-injured ipsilateral side of the hippocampus. Then, it may be concluded that contrary to what has been assumed previously, the endogenous spontaneous neurogenesis is insufficient for replacing the lost neurons and to achieve global repair of neonatal brain injured by HI.

The present study demonstrates that despite the severity of brain pathology induced by neonatal HI, administration of sodium butyrate for 5 days following the onset of the insult appeared to decrease the cerebral damage by preventing severe atrophy or brain asymmetry. Our findings are in general agreement with those reported previously that histone deacetylase inhibitors (VPA, TSA and SB) exhibit a neuroprotective effect in cerebral injury induced in adult rodents [13, 14, 16]. Thus, based on the beneficial effects proven in the above studies, the potential use of HDACis for the mobilization of endogenous progenitors seemed to be reasonable as a therapeutic avenue for restoring the damaged neonatal brain. Yet, only a few papers have presented neuroprotective action of these agents in the immature brain and those addressing the impact of HDAC inhibitors upon post-injury neurogenesis remain particularly limited [28–31]. Moreover, the aims of the mentioned above studies do not always include the assessment of maturation of the proliferating cells into neurons and other cell types.

We assumed that the reduced deficit of weight in the hypoxic-ischemic hemisphere after SB administration reflects compensatory formation of new cells. Surprisingly, it occurred that SB did not enhance the population of dividing cells in DG. As was mention above, it may be due to a maximal rate of cell proliferation in the chosen stage of brain development and then, it cannot be upregulated any further by epigenetic manipulation. Otherwise, exposure to SB restored the lost neuroblasts in the ipsilateral SGZ 14 days after HI. This finding raises the possibility that new cells will participate in the recovery by supplementing new neurons. This prediction was reinforced by the fact that neuroblasts express DCX, a marker with multiple beneficial functions including migration, differentiation, and survival. However, in contrast to our expectation, the number of newly generated cells positive for both signals, BrdU and calbindin, present throughout the granular cell layer, did not return to the control level, despite the apparently robust production of immature neurons after exposure to SB.

At present a clear picture of the critical initial signaling events responsible for the insufficient neurogenesis in the presence of SB remains elusive. One of the most likely explanations is that the injured SGZ does not provide an environment that is conducive to the maturation or survival of the newly formed neurons, which is in agreement with other published data [8, 51, 52]. It is supposed that an anti-neurogenic environment counteracts maturation of neuroblasts and may contribute to the predominant glial fate in vivo, much like in the adult brain [53]. Thus, it follows, that in our experimental conditions, the expected SB-induced regenerative capacity fails as a source of meaningful compensation for lost neuronal circuits. According to the results of the detailed elegant study performed by George et al. [29], the impact of HDAC inhibitors upon post-stroke neurogenesis is likely to depend on many factors, including the age of the animal at the time where neurogenesis is assayed, duration of HDAC inhibition before the BrdU labeling, and/or stage of the evolution of injury. Our results remain in disagreement with reports indicating that SB, as well as other inhibitor treatments, induced neuronal cell differentiation in brain areas injured by ischemia and may contribute to long-term beneficial effects in adult rodents [13, 16].

Neonatal hypoxic-ischemia led to marked reduction in the number of oligodendrocyte progenitor cells (OPCs; BrdU/NG2), pronounced in the ipsilateral hippocampus. This response remains in agreement with already published data showing susceptibility of oligodendrocytes to neonatal HI injury [6, 54, 55]. OPC depletion may lead to impairment of oligodendrocyte maturation at a premyelinating developmental stage and in consequence to white matter damage [56]. However, the markers attributed to more advanced stages of oligodendrocyte maturation (BrdU/O4-positive cells) seem to be similarly distributed in control and injured animals at 4 weeks after HI. It might be due to ongoing compensation by the active gliogenesis process which is known to proceed most intensely during both the perinatal period and the first postnatal weeks in rats [57–59]. Since OPCs are supposed to be generated in excess during ontogenesis, the number of mature oligodendrocytes is often reported to be normal in spite of their decreased number after insults [60, 61]. This implies the protection of myelin formation which is still in active state in neonates [62].

Post-insult treatment with SB-histone deacetylase inhibitor robustly increased the number of OPCs in both hippocampi at 2 and 4 weeks of recovery reinstating the physiological pool of OPCs, which could contribute to sustain endogenous homeostasis. The present data are consistent with findings from in vitro studies pointing to the role of SAHA, another HDACi, in preserving mature oligodendrocytes in the mouse optic nerve after OGD [63]. On the other hand, Fleiss et al. [28] using a model of lipopolysaccharide-sensitized hypoxia-ischemia found that trichostatin A, which is also a common inhibitor of HDAC, did not affect the insult-reduced oligodendrocyte number, what could be observed by Olig-2 staining. The obvious differences could be due to different investigation models. It is worth to point out that there are also interesting but intriguing data indicating that just HDAC activity is essential for oligodendrocyte differentiation in the developing rodent brain [64–68]. Of note, activity of class I histone deacetylases (HDAC1 and HDAC2) is required to regulate oligodendrocyte differentiation and maturation by reducing the expression of co-repressors of myelin gene transcription [68, 69]. Furthermore, genetic ablation of both isoforms in the mouse blocks these processes [68]. However, it seems rather hard to compare the study conducted by Ye et al. [68] in non-pathological experimental conditions with hypoxic-ischemic brain injury performed in our study. Therefore, considering our data we cannot negate the role of HDAC inhibition in mediating the effect observed in the course of our investigation.

One of the interesting findings obtained in the current work is that the protective effect of SB on oligodendrocyte progenitors seen in the ipsilateral hemisphere 2 weeks after the insult was associated with reduction of HI-induced microglial cells and infiltration of macrophage/monocytes (ED1-positive). It might be one of the potential mechanisms whereby SB protects oligodendrocytes. This is generally consistent with reported earlier correlation between oligodendrocyte protection and suppression of the proinflammatory action of microglia/macrophages in adult rodents [15, 70, 71]. Convincing evidence showed that in some conditions HDACis induced microglial apoptosis to protect against neuronal death [72]. The effects of HDAC inhibition described above may also, in addition, improve the environmental milieu surrounding nearby neurons and indirectly exert a neuroprotective effect.

At present we cannot outline precisely the molecular mechanism(s) directly linked to the observed beneficial effects of sodium butyrate after neonatal HI. One, most probable scenario, is the increased acetylation of histones which is a virtue of HDAC inhibitory activity. Indeed, HDACi treatment has been reported to be associated with marked upregulation of AcH3 in the DG after ischemia in adult rodents [14, 16]. In our study SB application elicited an increase in acetylated histone H3 level only in sham (control) animals. In contrast to others, the same mode of SB application after hypoxia-ischemia did not result in the elevation of histone acetylation above the vehicle control value. The unchanged acetylation after HDACi injection was also described by [28] in male mice in a model of LPS/HI. The unexpected lack of HDACi effect could be due to stress response of post-injured tissue which temporarily induced inhibition of histone acetyltransferase activity and/or overactivation of HDAC during this stage of development. This prediction may be reinforced by data reported by Sandner et al. [31] showing prolonged (lasting 4 months) overactivation of HDAC after hippocampal lesioning. If such an event took place during the time of our assay, it may account, at least partially, for the lack of SB influence. On the other hand, it is also possible that the evolving inflammatory response to HI injury may temporarily suppress HDAC activity producing a lack of response to inhibitors [73]. Nevertheless, our results, independent of the above considered mechanism, may reflect a novel equilibrium reached between acetylation and deacetylation processes in order to maintain the proper gene transcription. We can state that the effects of sodium butyrate treatment do not seem to be achieved through increased acetylation of histone H3. Histones are not the sole substrate of HDACs. Several of histone deacetylase isoforms have been shown to regulate acetylation of a plethora of non-histone proteins, and thus, we cannot rule out the possibility that the neuroprotective effect of HDAC inhibitors are multifold and linked to influencing a diverse array of targets [24, 27].

One of the HDACi targets known to be involved in neurogenesis and survival of newborn cells after ischemic insult is BDNF [74, 75]. HDAC inhibition activates BDNF promoter IV and increases BDNF mRNA levels in dissociated rat cortical neurons [76]. The increased BDNF level in the ipsilateral hemisphere found in our study after SB treatment may imply that trophic support plays a role in neurogenesis. This hypothesis has been intensified by reports showing that administration of endogenous BDNF after HI in neonates decreased brain damage [77]. Furthermore, the role of BDNF-TrkB signaling in mediating HDACi-induced cell proliferation and differentiation in the ischemic adult rodents was supported by several findings [13, 71]. Thus, at least, this mechanism mediated by BDNF underpinning neuroprotection after SB treatment in neonates is supposed to be one of those commonly reported in adult cerebral studies. To define the role of BDNF, further studies are needed.

One would expect that the neuroprotective/neurogenic effects of SB found in our study after HI induced in neonatal rats would be further translated to improving the neurobehavioral performance. To this end, we employed sensorimotor as well as cognitive tests to assess motor coordination and memory deficits in animals after HI, treated and untreated with SB. It was expected that by the time of testing (P33–P83), newly generated neuroblasts became fully matured. In contrast, their number still remained below control level in the ipsilateral side after HI as well as after SB treatment. Only in two of the five individual tests performed (MWM and USV recording), HI-injured rats showed significant behavioral deficiency when compared to controls. Mildly impaired cognitive function observed in other tests performed indicate that HI-treated rats showed some degree of recovery as it has also been suggested by previous studies [78, 79]. Administration of the histone deacetylase inhibitor after the onset of the insult did not counteract significantly the functional brain impairments caused by HI; however, there was a tendency for improving learning, as is seen in graph presenting the results of MWM. In spite that SB protected against ischemia-induced damage of brain structures contributing to several cognitive processes [80, 81], the remaining number of new-generated mature neurons probably occurred to have insufficient potential for the compensation of interplay between hippocampus and prefrontal cortex. Thus, the rescue of neurobehavioral deficits needs restoration of the granule cell layer and preservation of the neural networks. Our data are similar to those reported by Sandner et al. [31] showing that HDAC inhibitor injected for as long as 7 weeks was sufficient to alleviate some, but not all behavioral impairments due to neonatal ventral hippocampal lesioning. However, in the above-mentioned experiment, there was a clear discrepancy between injury progression and behavioral outcome. In contrast to our finding, in adult rodent models of cerebral injury treated with HDACis, functional improvement correlated steadily with neurogenesis and reduced severity of neurological injury [13–15, 17]. Consistent with these findings, many studies have shown that experimental reduction of adult neurogenesis impairs hippocampal memory formation and conversely, stimulated neurogenesis seems to be one approach to enhance cognitive recovery. On the other hand, contradictory accounts showed no impact of adult brain neurogenesis reduction on memory formation, where the role of newly generated neurons has previously been strongly suggested [43, 82, 83]. In the view of this inconsistent data, a stronger link between increased neurogenesis in the injured brain and functional outcome after treatment with HDACis needs to be pursued with future work.

*In conclusion*, the observed protective/neurogenic effect, following sodium butyrate (SB) treatment in neonatal rats subjected to hypoxia-ischemia, is associated with a reduction of brain infarct. It may be due to restoration of hypoxia-induced loss of neuroblasts and oligodendrocyte progenitors as well as suppression of microglial cells. In contrast, the number of new mature granule cells did not reach the control level, which indicates a limited effect of SB on this stage of neurogenesis. SB did not appear to improve neurological outcome; it may be deduced that in our experimental condition the insufficient number of cells within the granular cell layer observed after SB treatment does not lead to full compensation for the lost neuronal circuits. Knowledge about the molecular mechanisms of SB action is still in early stage of discovery. Continuing research along this line may provide a better understanding of responses to the inhibition of histone deacetylases in the neonatal hypoxic-ischemic brain.
